# Toward Wireless Implantable Robotic Systems Driven by Magnetic Field for Personalized Therapy

**DOI:** 10.1002/adrr.202500077

**Published:** 2025-07-27

**Authors:** Yusheng Wang, Ruijian Ge, Xiaoguang Dong

**Affiliations:** 1 Department of Mechanical Engineering, Vanderbilt University, Nashville, USA; 2 Department of Biomedical Engineering, Vanderbilt University, Nashville, USA; 3 Vanderbilt Institute for Surgery and Engineering, Vanderbilt University, Nashville, USA

**Keywords:** implantable device, magnetic actuation, magnetic materials, wireless sensing

## Abstract

Robotic materials are playing an increasingly important role in enabling sensing and actuation at small scales. Recent advances have shown that these materials can dynamically respond to environmental cues while supporting remote sensing for versatile applications particularly healthcare. Among them, magnetically responsive materials such as magneto-elastic and magnetoelectric materials, offer compact, wireless solutions for miniaturized actuators, sensors, and energy transmitters, with significant potential in personalized medicine. However, key challenges remain in integrating magnetic materials toward implantable robotic systems, in achieving miniaturization, biocompatibility, and closed-loop therapy. This perspective highlights recent developments in magnetic materials and magnetically actuated devices for wireless sensing, actuation, and energy harvesting, toward implantable robotic systems for closed-loop therapy. We survey magnetic materials in enabling pumps, valves, and other drug delivery modules and evaluate their performance in terms of actuation field, biocompatibility, and applicable locations. Additionally, we also survey their sensing functions when integrating with other stimuli-responsive materials for different physiological conditions as well as energy harvesting functions for powering. Finally, we discuss future directions in miniaturization, safety, and long-term in vivo stability to facilitate clinical translation. This work provides a forward-looking perspective on next-generation, minimally invasive, robotic implantable systems for personalized disease monitoring and therapeutic intervention.

## 1 | Introduction

Miniaturized wireless medical implants [1–4] are crucial due to their ability to enable minimally invasive procedures, which reduce tissue damage, complications, and recovery time. They enhance patient comfort and quality of life by being less obtrusive and allowing greater freedom of movement, as wireless designs eliminate external wires. These implants facilitate continuous monitoring and remote data transmission, allowing for real-time health tracking and better management of chronic conditions. They enable targeted and efficient treatments, delivering localized therapy that minimizes side effects and supports precision medicine. Wireless implants reduce infection risk by avoiding external components, and advances in power solutions like inductive charging or other type of energy harvesting extend their lifespan. Their compact size allows access to hard-to-reach areas, making them ideal for complex organs such as the brain [2] and heart [5]. Improved biocompatibility reduces immune response and inflammation, making these devices safer for long-term use and easier to remove or replace. Recent advances in various stimuli-responsive materials allow for minimally invasive procedures and enhance device efficiency by their stimuli-responsive behaviors [6, 7]. Their integration into medical implants improves patient outcomes by providing precise, noninvasive monitoring and treatment, making them essential for the future of personalized medicine and long-term healthcare solutions.

Among various stimuli-responsive materials, magnetic composite materials [8, 9] are particularly promising for enabling miniaturized medical implants. They are formed by integrating traditional magnetic particles with other substances such as elastic or electrically active materials [10, 11]. These composites allow remote actuation through external magnetic fields without direct interaction with surrounding biological tissues. One notable example is magnetoelastic materials [11], which are composites embedded with magnetic particles that deform under external magnetic fields. These materials have demonstrated their utility as remotely controlled actuators [8, 9, 12, 13], functioning as pumps and valves for drug delivery and sampling [14]. Beyond their mechanical deformation capabilities, magnetoelastic materials also show significant potential for wireless sensing of physiological properties. Their elastic response to external magnetic fields can be harnessed to monitor critical changes in the body, such as tissue stiffness [15], fluid viscosity [16], and fluid flow [17]. A key advantage of these materials is their ability to operate without physical connections, allowing for extreme miniaturization that is ideal for use in delicate, hard-to-reach areas. Another important magnetic material is magnetoelectric material [18], which combines magnetic and electric properties. These materials not only detect physiological changes but also generate electrical signals in response to magnetic fields, enabling both remote power delivery and wireless data transmission. This is particularly advantageous for implantable or swallowable devices [19, 20]. For example, in gastrointestinal (GI) applications, magnetoelectric sensors can be integrated into ingestible capsules that monitor internal conditions and wirelessly transmit data to external receivers.

The combination of magnetic materials with other smart materials has opened further opportunities for multifunctional sensors, capable of both sensing and actuation. For instance, hybrid materials that integrate magnetic components with responsive polymers can adapt to body conditions while transmitting data through magnetic signals. These combinations, however, introduce new challenges, such as ensuring long-term biocompatibility. Some magnetic materials, while efficient in data transmission or power generation, may degrade over time or cause adverse biological responses. To address this, researchers are developing coatings and composites that preserve the functional properties of magnetic materials while making them safe for long-term use in the body. The integration of magnetic composite materials in biomedical smart devices offers tremendous potential for creating compact, wireless, and responsive sensors. These sensors can function in a variety of physiological environments, providing real-time data on internal conditions and offering remote actuation capabilities. However, careful consideration of biocompatibility, degradation, and long-term stability is essential for their successful application in clinical settings [21]. Developing compact and wireless sensors that can be reliably implanted to monitor such conditions continuously remains a significant challenge.

**FIGURE 1 |:**
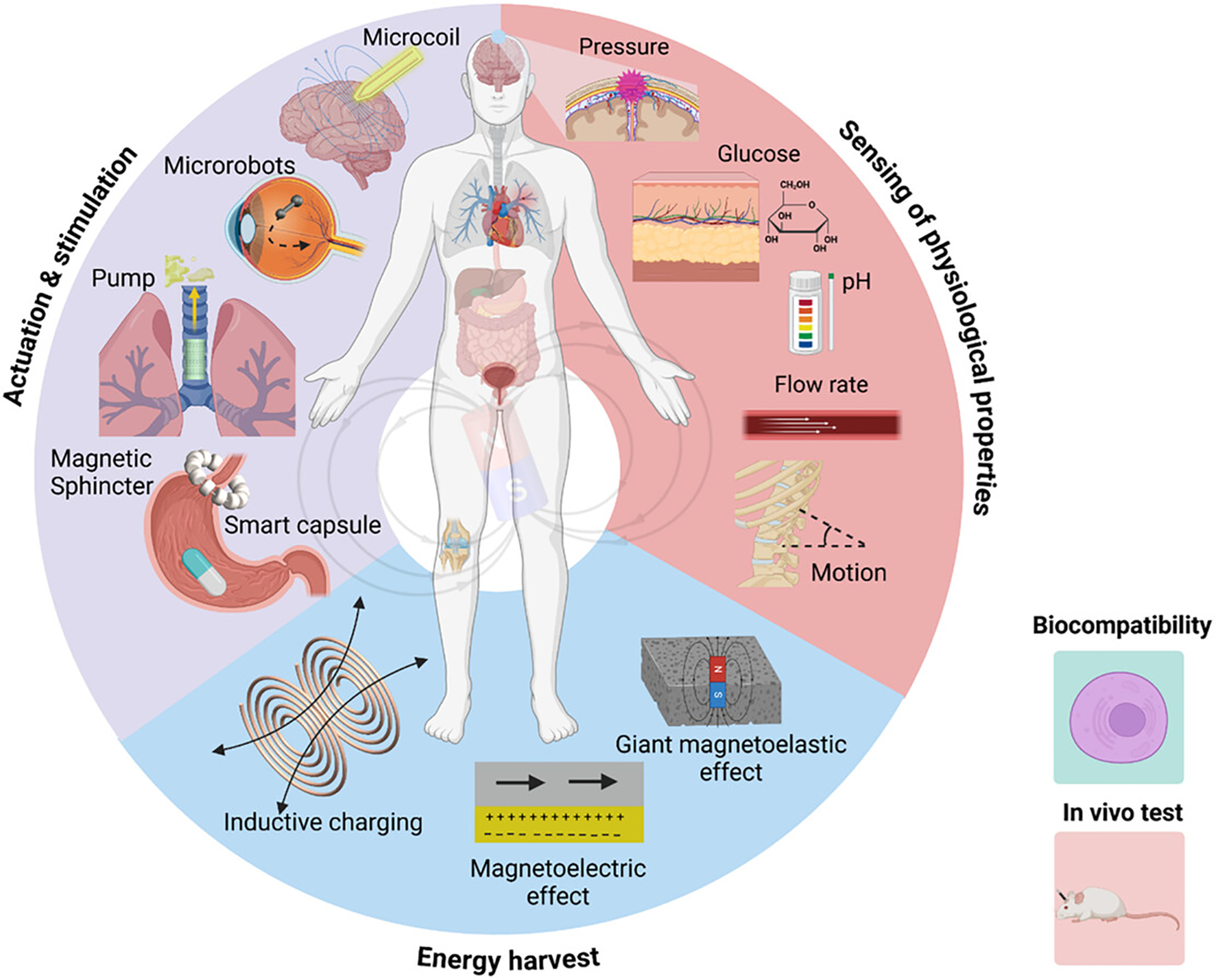
Overview of magnetic composite materials for implantable devices including actuation and stimulation, sensing, and energy harvesting. Created via Biorender.com.

This review highlights the latest advances toward robotic implantable devices and systems by introducing recent works on implantable devices enabled by magnetic materials and driven by magnetic fields (Figure 1). The review focuses on the pivotal role of magnetic materials and magnetic fields in enabling wireless sensing and stimulation in implantable devices. We survey the performance of magnetic materials in enabling pumps, valves for drug delivery, and other applications. Meanwhile, we examine the performance of these materials in terms of sensing speed, spatial resolution, and penetration depth. We compare the communication abilities of these sensors under different physiological conditions, addressing their effectiveness in various regions of the body, where factors like tissue density or fluid content may influence sensor performance. These innovations are paving the way for the next generation of minimally invasive medical technologies, offering unparalleled precision and control in the monitoring and treatment in various health conditions.

## 2 | Overview of Magnetic Materials for Implantable Devices

Common magnetic particles, such as iron, magnetite, cobalt, and nickel, exhibit magnetic properties like ferromagnetism, ferrimagnetism, or superparamagnetism [22], making them essential for a wide range of applications (Table 1). These particles are used in sensors, biomedical devices, data storage, and electromagnetic shielding. Advanced materials like neodymium–iron–boron (NdFeB) are strong permanent magnets used in high-performance applications, while iron oxide nanoparticles are valued for their superparamagnetic properties in targeted drug delivery and Magnetic Resonance Imaging contrast agents. Some nanoscale ferromagnetic or ferrite materials can exhibit hard magnetic properties [32], characterized by high coercivity and remanence. These properties arise from factors such as size-induced magnetic anisotropy, interparticle interactions, and surface effects, enabling the materials to retain magnetization even after the external magnetic field is removed. As a result, such nanoscale materials are promising for applications in data storage, permanent magnets, and spintronic devices. On the other hand, ferrites, such as manganese–zinc ferrite, are useful in electronic components for their high resistivity and low power loss. The versatility of these particles makes them critical for both industrial and biomedical technologies.

Magnetic particles can be blended or synthesized with various chemicals to create functional materials. The concentration and size of these magnetic particles not only influence the material’s response to an external magnetic field but also alter properties such as stiffness and stretchability, necessitating careful balance, and optimization in design. For instance, in magnetoelastic materials, a higher concentration of magnetic particles enhances the response to a specific magnetic field, while also increasing stiffness and reducing stretchability. A smaller particle size is usually preferred as it can be mixed or synthesized better with other materials. However, smaller particles typically require complex synthesis or milling process. When the particle size scales down, the magnetic properties may also change such as the magnetic coercivity.

Magnetic materials are crucial for implantable devices because they enable wireless communication, remote actuation, and power transfer, which are essential for creating minimally invasive, efficient, and reliable medical implantable devices. Their ability to respond to external magnetic fields allows for noninvasive control and monitoring of devices inside the body, eliminating the need for wired connections for powering the implant or surgeries to adjust the device. For instance, magnetically actuated devices can perform tasks like drug delivery, tissue stimulation, or movement through the body, while magnetic sensors can monitor physiological parameters such as pressure, blood flow, or tissue stiffness. Additionally, magnetic materials used in wireless power transfer systems can recharge implants, such as pacemakers or neurostimulators, without the need for battery replacement surgeries. The biocompatibility and compactness of specific magnetic materials also make them suitable for use in confined or sensitive areas, enhancing the functionality and longevity of implantable medical devices.

Magnetoelastic materials are smart materials that exhibit a coupling between magnetic and elastic properties, meaning they deform in response to external magnetic fields and change their magnetic properties when subjected to mechanical stress [12]. This unique characteristic allows them to be used for wireless sensing and actuation, making them highly suitable for applications like implantable medical devices. They can be miniaturized and used to monitor physiological conditions such as tissue stress or blood flow, or for remote actuation in devices like stents or drug delivery systems, all without the need for physical connections. Their compactness and wireless functionality make them ideal for use in hard-to-reach areas within the body.

## 3 | Magnetic Materials for Actuation in Implantable Devices

Magneto-elastic materials have been shown to undergo controlled deformation when actuated by external magnetic fields [12, 13]. By tailoring the magnetization profiles of these composite materials, precise and programmable motions can be achieved under varying magnetic field conditions. Such magnetic actuation has been harnessed in implantable devices for targeted therapeutic delivery and mechanical stimulation (Table 2).

**FIGURE 2 |:**
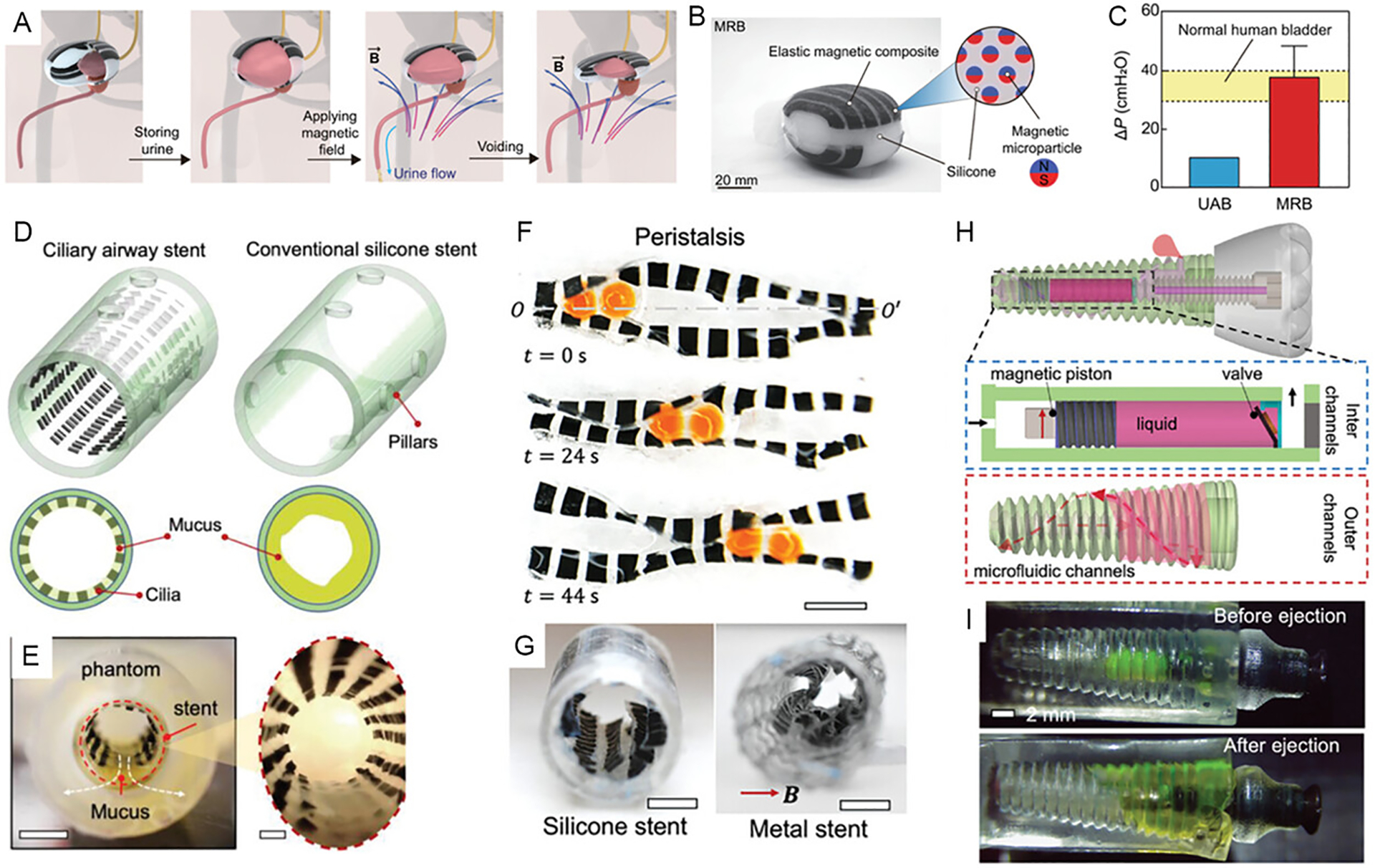
Implantable devices with magnetic pumps and valves. (A) Magnetic actuator for facilitating voiding of the bladder. Reproduced with permission [36]. Copyright 2022, AAAS. (B) Image of the magnetic actuator. Reproduced with permission [36]. Copyright 2022, AAAS. (C) Pressure of the magnetic actuator when being actuated. Reproduced with permission [36]. Copyright 2022, AAAS. (D) Magnetic cilia for pumping mucus in an airway stent. Reproduced with permission [34]. Copyright 2023, Wiley Group. (E) Image of mucus transportation by the magnetic cilia [34]. Copyright 2023, Wiley Group. (F) Magnetic peristaltic pump for transporting solid cargos. (G) Esophageal stent-integrated magnetic peristaltic pumps. Reproduced with permission [33]. Copyright 2024, Wiley Group. (H) Magnetic dental implant with on-demand liquid release. Reproduced with permission [23]. Copyright 2024, Wiley Group. (I) Image of liquid ejection by a dental implant with magnetic pump and valve. Reproduced with permission [23]. Copyright 2024, Wiley Group.

Pumps, for instance, are critical components in implantable systems for delivering therapeutic agents such as drugs. Magnetically actuated pumps offer a wireless and remotely controllable solution for liquid propulsion (Figure 2). Yang et al. (2022) [36] developed an implantable magnetic soft robotic bladder capable of applying compression to assist urination in patients with underactive bladder conditions (Figure 2A–C). For airway applications, Wang et al. (2023) [34] designed an artificial ciliary airway stent to transport excessive mucus within the trachea (Figure 2D,E). The artificial cilia, composed of magnetic composites, are wirelessly actuated to achieve minimally invasive mucus clearance. In the GI tract, Sharma et al. (2024) [33] introduced a stent-based magnetically actuated soft undulating pump capable of transporting both viscous fluids and solid cargo, addressing esophageal dysmotility (Figure 2F,G). Additionally, Lee et al. (2017) [35] demonstrated a batteryless, fully implantable insulin pump actuated by an external magnetic field, allowing for precise dosage control through repeated magnetic stimulation.

Controlling fluidic functions within implants is essential for achieving precise, programmable therapeutic delivery. Magnetically actuated valves have been developed to enable reliable and repeatable control over the opening and closing of fluidic channels. For instance, Pereira et al. (2023) [39] introduced a miniature glaucoma implant capable of adjusting intraocular pressure through a magnetically controlled valve. Similarly, magnetic sphincters have been reported that utilize magnetic interactions to maintain lumen closure without mechanical components [50]. Xu et al. (2024) [23] demonstrated a wireless miniature pump and valve system embedded in dental implants that allows remote-controlled liquid delivery, with a storage capacity of up to 52 μL (Figure 2H,I). In ophthalmic applications, Wang et al. (2018) [38] proposed an intravitreal implantable magnetic micropump integrated with a micro check valve for on-demand delivery of vascular endothelial growth factor (VEGF)-targeted therapeutics.

**FIGURE 3 |:**
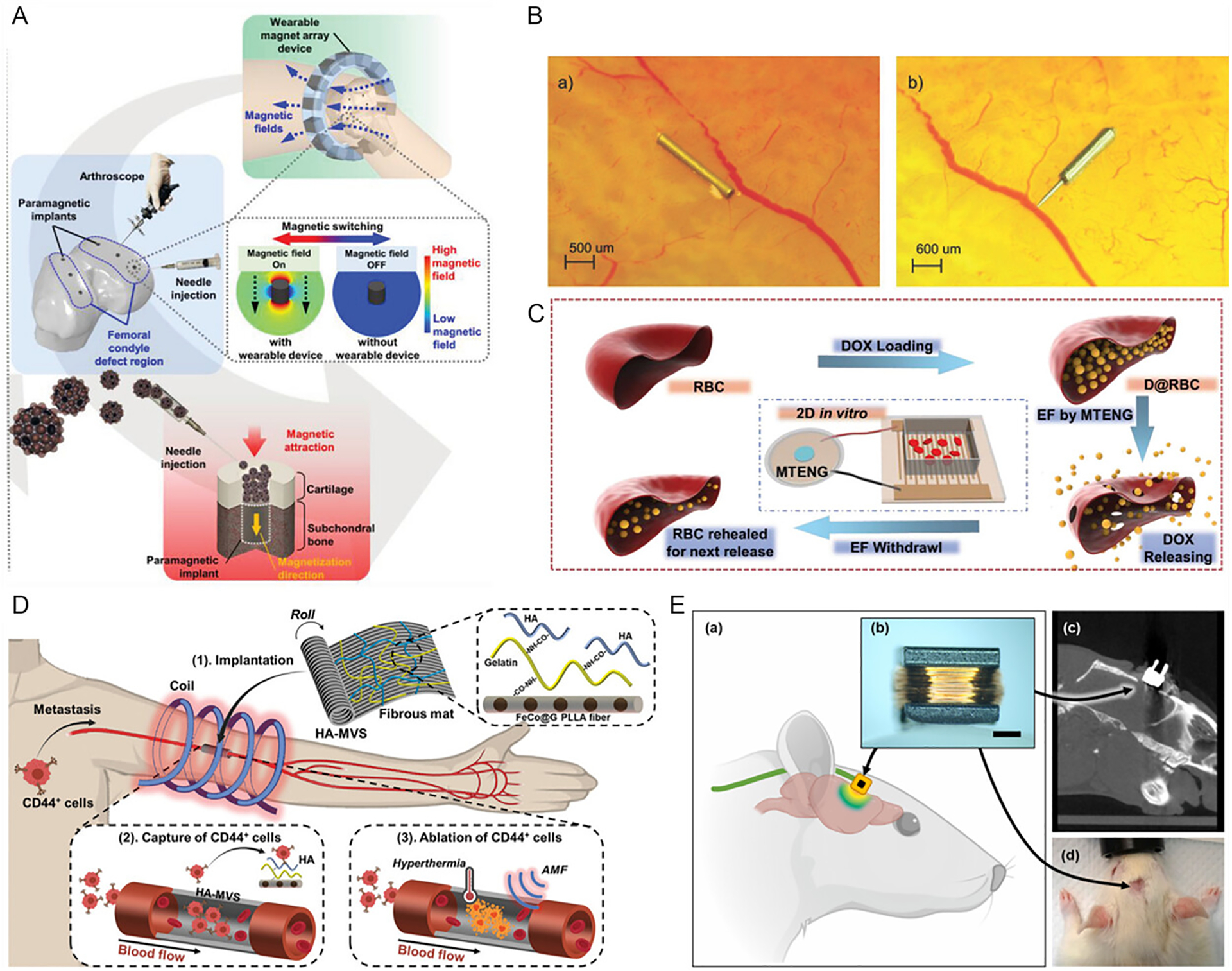
Implantable devices with magnetic materials enabled therapeutic functions. (A) A magnetic implant system consisting of microcarriers, a portable magnet array device, and a paramagnetic implant. Reproduced with permission [42]. Copyright 2021, Wiley Group. (B) A microrobot for drug delivery inside the eye. Reproduced with permission [43]. Copyright 2015, Elsevier. (C) A magnetic triboelectric nanogenerator for drug release. Reproduced with permission [41]. Copyright 2019, Wiley Group. (D) An integrated trapped device with wireless magnetothermal response to remove tumor cells. Reproduced with permission [45]. Copyright 2022, Wiley Group. (E) An implantable coil to induce neuromodulation effects. Reproduced with permission [49]. Copyright 2021, Wiley Group.

Drug delivery can be achieved through the coordinated use of magnetically actuated pumps and valves, as well as embedded magnetic particles that enable alternative release mechanisms. These functionalities have been demonstrated across a range of implantable devices, typically relying on direct current (DC) magnetic fields for mechanical injection or alternating current (AC) magnetic fields for induction heating-based thermal release. For example, Go et al. (2021) [42] introduced a multifunctional magnetic implant system composed of magnetic microcarriers, a portable magnet array, and a paramagnetic implant (Figure 3A). This platform enables targeted delivery, secure fixation, and magnetically induced stem cell differentiation. Chatzipirpiridis et al. (2015) [43] demonstrated an implantable magnetic tubular microrobot for ocular drug delivery, which fits within a 23-gauge needle for minimally invasive, sutureless injections (Figure 3B). Zhao et al. (2019) [41] developed an implantable magnetic triboelectric nanogenerator capable of producing up to 70 V post-implantation, enabling drug release and cancer therapy (Figure 3C).

Magnetic hyperthermia based on hysteresis-induced heating has also been utilized in implantable systems for drug delivery and cancer treatment. Yin et al. (2022) [45] presented a vascular-like implant that traps circulating tumor cells and responds wirelessly to magnetothermal stimulation (Figure 3D). Sasikala et al. (2015) [46] developed a magnetic nanofiber implant for endoscopic hyperthermia and tumor-triggered drug release. Zheng et al. (2023) [44] designed an implantable, magnetically actuated capsule paired with a portable actuator to enable on-demand wireless drug delivery. Wang et al. (2019) [47] explored the inductive heating properties of eutectic gallium-indium (EGaIn) alloys under AC magnetic fields for drug release and thermochemotherapy. Additionally, Yang et al. (2022) [37] introduced an implantable magnetic microactuator capable of breaking down obstructive blood clots within catheters. Li et al. (2024) introduced ferromagnetic liquid robots for cleaning thrombus in blood vessels wirelessly [51].

Finally, electrical stimulation represents another important therapeutic modality in implantable devices, with magnetic materials playing a key role primarily through energy harvesting mechanisms. Neurological stimulation has been achieved using implantable coils or magnetoelectric materials to deliver localized brain stimulation wirelessly. For example, Lee et al. (2024) [49] demonstrated the feasibility of magnetic stimulation via an implantable coil capable of inducing neuromodulatory effects (Figure 3E). In earlier work, Lee et al. (2016) [48] introduced a novel microcoil design that effectively activated cortical neurons and elicited behavioral responses. Yu et al. (2020) [52] presented a magnetoelectrically powered, untethered, and programmable neural stimulator with a remarkably low static power consumption of just 23.7 μW, enabling efficient long-term operation. Additionally, Sivaji et al. (2019) [53] reported a wireless pulse generator for nerve stimulation, powered by arrays of small metallic coils, further underscoring the versatility of magnetic energy harvesting for implantable neuromodulation systems.

In summary, magnetic material-based actuation and stimulation are fundamental components of implant robotic systems. Unlike traditional actuation or stimulation methods, magnetic material-based systems can operate wirelessly, allowing for untethered and minimally invasive interventions. This technology eliminates the need for an energy storage module, such as a battery, within implantable devices, thereby overcoming limitations related to lifespan, size, and safety. Overall, the advantages of magnetic-based devices highlight their significant potential for application in more confined regions of the human body, facilitating targeted minimally invasive surgeries and promoting long-term recovery.

## 4 | Magnetic Materials for Sensing in Implantable Devices

Magnetic materials integrated into implantable devices enable a wide range of sensing applications, including position tracking, force and pressure monitoring, and temperature sensing which are essential for real-time diagnostics and therapeutic feedback (Table 3). These materials also facilitate the monitoring of mechanical strain, offering valuable insights into tissue healing processes, particularly in load-bearing scenarios such as bone repair. Furthermore, magnetic materials support wireless communication and power transfer, eliminating the need for wired connections. This not only reduces the risk of infection but also enhances patient comfort and mobility, making them well-suited for long-term physiological monitoring and data transmission in clinical settings.

**FIGURE 4 |:**
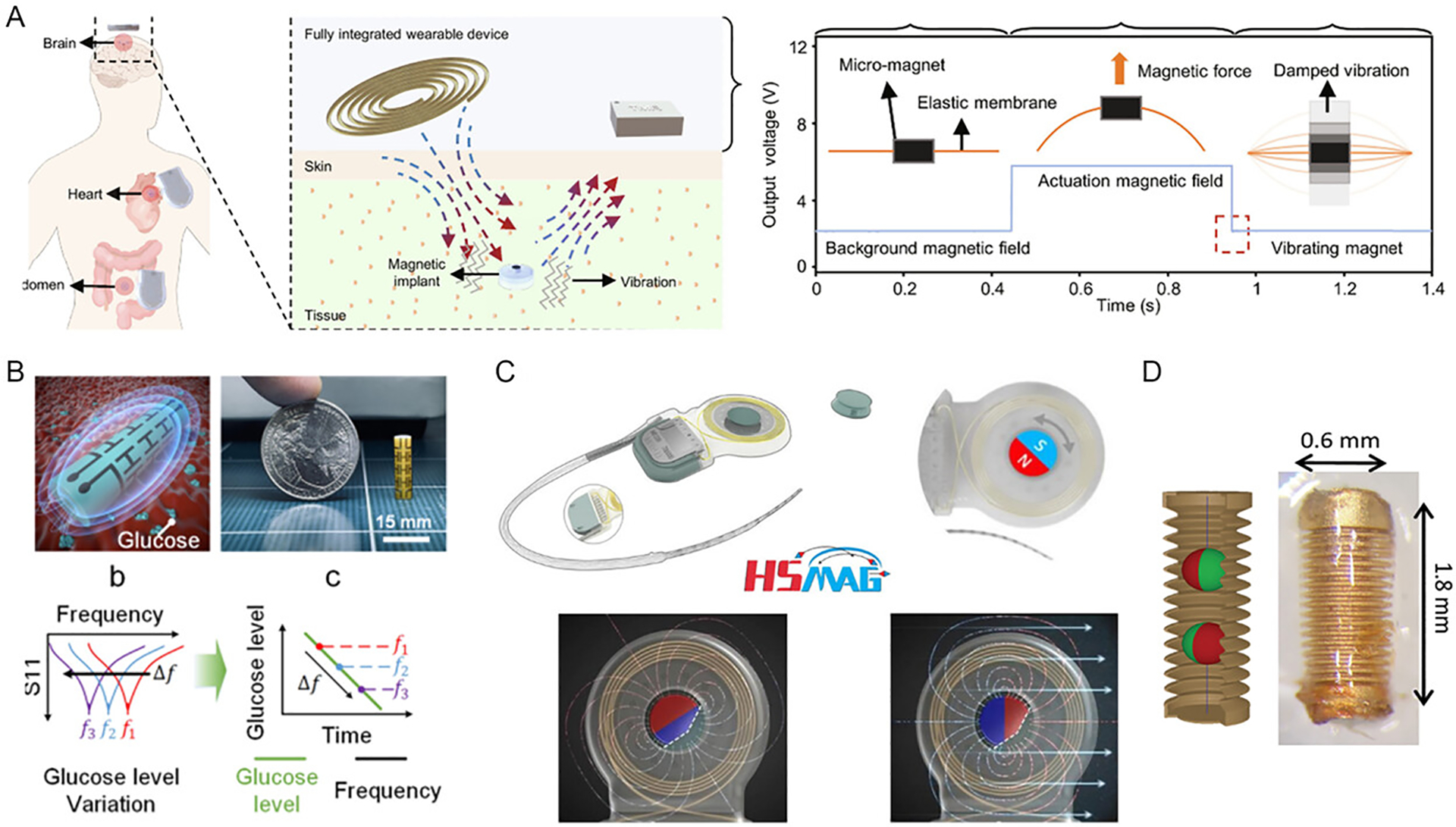
Implantable devices with magnetic resonance-based sensing. (A) A set of miniature magnetic implants for measuring cerebrospinal fluid viscosity, intracranial pressure, and cerebrospinal fluid glucose. Reproduced with permission [56]. Copyright 2024, AAAS. (B) An electromagnetic-based sensor for blood glucose level monitoring. Reproduced with permission [62]. Copyright 2022, Springer Nature. (C) A cochlear implant magnet to hold the headpiece in place. Reproduced with permission [63]. (D) A resonant magneto-mechanical sensor which can be used for subcutaneous pressure monitoring. Reproduced with permission [64]. Copyright 2023, AAAS.

Low-frequency magnetic field and magnetic sensor can also be used for sensing (Figure 4). For example, Wan et al. (2024) [56] reported a system composed of miniature magnetic implants paired with a wearable device to measure cerebrospinal fluid (CSF) viscosity, intracranial pressure, and glucose levels (Figure 4A). Kim et al. (2022) [62] proposed a subcutaneously implantable electromagnetic sensor that monitors dielectric permittivity changes associated with blood glucose levels (Figure 4B). Additionally, Ling et al. (2011) [61] employed magnetic resonance relaxometry to continuously monitor fluctuations in three clinically relevant cardiac biomarkers.

Resonance-based sensing is a widely adopted technique in implantable devices for monitoring physiological parameters. An LC circuit consists of an inductor (L) and a capacitor (C) arranged in series or parallel, forming a resonant system. At a specific resonant frequency, the inductive and capacitive reactances cancel each other, allowing the circuit to oscillate efficiently with minimal energy loss. Environmental changes such as variations in temperature, pressure, or chemical composition, affect the inductance or capacitance of the circuit, leading to a shift in the resonant frequency. This frequency shift can be wirelessly detected and correlated with physiological conditions. For example, Lin et al. (2024) [58] developed a robust hydrogel interface to improve the mechanical coupling between a wireless pressure sensor and biological tissues or organs, enhancing sensing fidelity. To allow sensor degradability, Boutry et al. (2019) [59] reported a fully biodegradable implantable pressure sensor capable of measuring arterial blood flow in both contact and noncontact modes, showcasing the potential of LC-based resonant sensors for transient and biocompatible monitoring applications. To allow distributed sensing, Herbert et al. (2022) [60] presented a fully implantable wireless system based on a multimaterial inductive stent capable of monitoring arterial pressure, pulse rate, and blood flow. In addition to LC circuits, Gleich et al. (2023) [59] also reported a wireless tracking mechanism based on magneto-mechanical resonators, addressing the challenges in tracking and sensing in medical applications (Figure 4D). In summary, these sensors operate without internal power sources, relying instead on passive magnetic coupling with an external reader, which minimizes implant size and eliminates the need for battery replacement.

**FIGURE 5 |:**
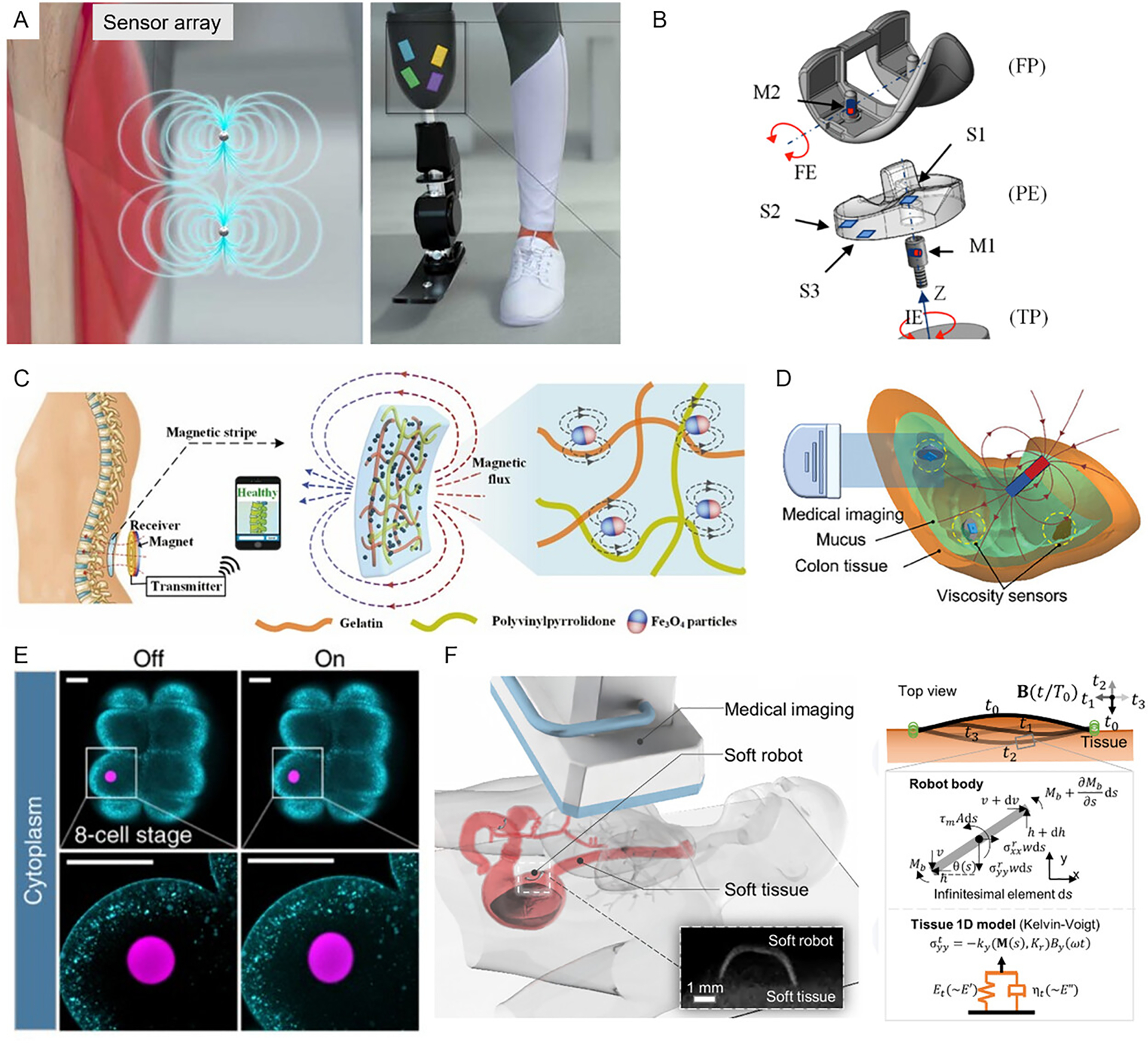
Implantable devices with magnetic materials for motion or shape-based sensing. (A) A magnetomicrometry-based structure to track human movement. Reproduced with permission [21]. Copyright 2022, Frontiers. (B) A magnetic measurement system to measure knee rotations in knee prostheses. Reproduced with permission [55]. Copyright, 2013 IEEE. (C) An implantable sensing system for real-time monitoring of bending of spines and joints. Reproduced with permission [54]. Copyright 2024, Elsevier. (D) A magnetic spinner with a magnetic-actuated soft robot for sensing mucus viscosity in situ. Reproduced with permission [16]. Copyright 2023, Wiley Group. (E) Magnetically responsive ferrofluid microdroplets for cellular mechanical property measurement. Reproduced with permission [29]. Copyright 2016, Springer Nature. (F) A wireless miniature soft robot controlled by a magnetic field to in situ sense the physiological properties. Reproduced with permission [15]. Copyright 2013, AAAS.

Magnetic field measurements have been utilized to track and detect movement within the human body. Taylor et al. (2022) demonstrated magnetic bead tracking for muscle contraction with high accuracy (Figure 5A). Arami et al. (2013) [55] introduced a magnetic measurement system designed for integration into smart knee prostheses, enabling precise detection of compound knee rotations (Figure 5B). Shi et al. (2024) [54] developed a wireless, battery-free, implantable sensing system for real-time monitoring of joint and spinal bending. The system utilizes a flexible magnetic strip implanted in the body, with signals detected by an external receiver (Figure 5C). Additionally, spintronics-based sensors represent another promising class of implantable magnetic devices, leveraging the spin of electrons to detect magnetic changes with high sensitivity [65]. Zhang et al. (2024) developed a 4D-printed electromagnetic architecture capable of stress detection, showing a great potential for biomedical applications [66].

Magnetic field-assisted/enhanced shape or motion-based sensing via medical imaging to assess advanced physiological properties of tissues and biofluids has been developed by researchers (Figure 5). Magnetic composites can exhibit distinct shape changes under identical magnetic fields depending on their interaction with local environments, especially when integrated with stimuli-responsive materials. These shape variations can be tracked using medical imaging modalities, providing enriched diagnostic information. For example, Wang et al. (2023) [15] developed a magnetic soft robot integrated with a mucoadhesive interface to adhere to GI tissue, enabling the *in situ* sensing of tissue viscoelasticity. Similarly, Xiao et al. (2023) [16] introduced a magnetic-actuated soft robot equipped with a spinner element to wirelessly sense mucus viscosity in the GI tract (Figure 5D). Beyond mechanical properties, electromagnetic-based sensors have been used to detect biofluid composition. Serwane et al. (2017) [23] utilized biocompatible ferrofluid microdroplets to achieve spatiotemporal measurements of tissue mechanical properties. (Figure 5E). Wang et al. (2023) [12] developed a magnetic soft robot to sense the adhesion, pH, and viscoelasticity on animal tissues tracked by X-ray or ultrasound imaging (Figure 5F).

In this section, we survey various applications of magnetic-based sensing in implantable devices. The unique properties of magnetic materials have expanded the possibilities for sensing applications, including the measurement of tissue stiffness and fluid viscosity, which can only be assessed through the active movement of the sensor. With this sensing capability, the system can effectively integrate with other treatment methods for timely and closed-loop interventions, thereby contributing to the development of a true robotic system that combines both actuation and control [67].

## 5 | Magnetic Materials for Energy Harvesting in Implantable Devices

**FIGURE 6 |:**
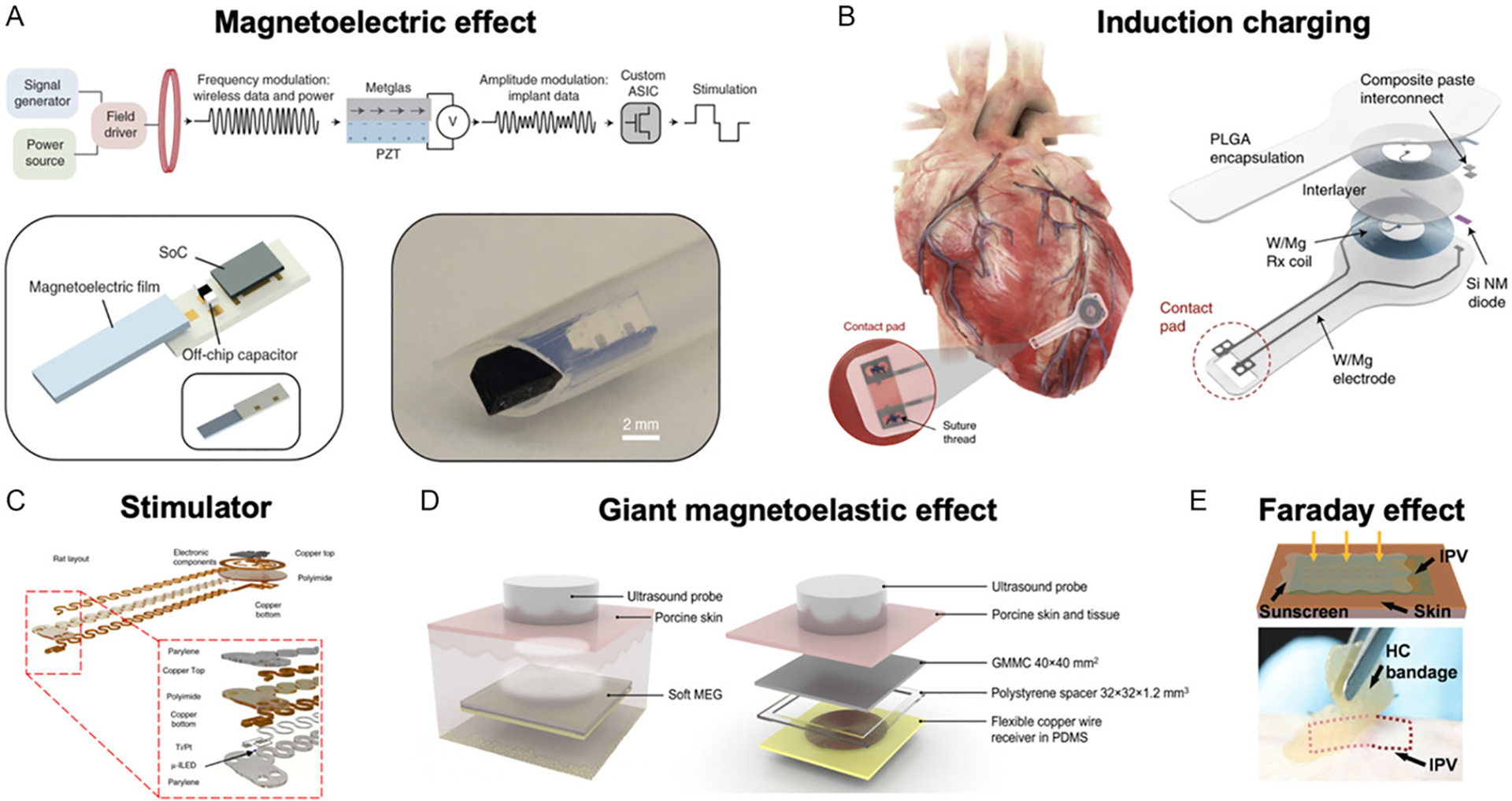
Implantable devices with magnetic materials for energy harvesting. (A) Miniature magnetoelectric-powered bio-implant for stimulating peripheral nerve. Reproduced with permission [68]. Copyright 2022, Springer Nature. (B) Fully bioresorbable, battery-free, implantable, leadless cardiac pacemaker. Reproduced with permission [69]. Copyright 2021, Springer Nature. (C) Wireless, battery-free, fully implantable pacemakers. Reproduced with permission [70]. Copyright 2019, Springer Nature. (D) Soft magnetoelastic generator for ultrasound excitation under tissue. Reproduced with permission [71]. Copyright 2021, Springer Nature. (E) Photovoltaic devices for powering in vivo medical device. Reproduced with permission [72]. Copyright 2016, Wiley Group.

Magnetic materials are widely used in energy harvesting systems for implantable devices by converting mechanical motion into electrical energy (Figure 6, Table 4). Movements such as muscle contractions, joint motion, or blood flow can cause a magnet to oscillate relative to a coil, thereby inducing an electric current that powers the implant. This approach is particularly advantageous as it leverages natural body motions, providing a sustainable and continuous energy source. Moreover, the harvested energy can also support wireless data transmission, enabling both power delivery and communication without the need for physical connections. For example, Mouzakis et al. (2008) [83] developed a self-powered microsensor based on MetGlas for diagnosing structural damage in implants. The system generates output signals that, when processed through a tailored algorithm, can identify implant damage without requiring external power sources.

The magnetoelectric effect is a promising mechanism for energy harvesting in implantable devices. Magnetoelectric materials convert external magnetic fields into electric fields, enabling wireless power transfer and data communication. These materials can be integrated into implantable sensors and stimulators to support fully wireless operation, especially in deep tissue environments where conventional wireless techniques face limitations. Magnetoelectric materials generate power in response to low-frequency magnetic fields, making them well-suited for applications such as wireless neural stimulation, deep-tissue sensing, and powering bioelectronic implants. For example, Chen et al. (2022) [68] developed a state-of-the-art miniature MagnetoElectric-powered Bio ImplanT (ME-BIT), which can be wirelessly powered several centimeters beneath the tissue surface and deliver sufficient energy for peripheral nerve stimulation (Figure 6A). Similarly, Yu et al. (2022) [73] created a 6.2-mm^3^, 30-mg implantable stimulator with an integrated system-on-chip (SoC) that leverages magnetoelectric mechanisms for robust, efficient wireless power delivery, programmable control, and synchronized stimulation.

Inductive charging represents another effective method for wirelessly powered implantable devices. Inductive charging relies on a dedicated external magnetic field, typically from a charging coil, to deliver higher, more controlled power for recharging batteries efficiently. While energy harvesting using ambient conditions is ideal for sustaining long-term, battery-free operation in remote or implantable devices utilizing all sorts of environmental energy, inductive charging is particularly suited for scheduled energy delivery in applications using highfrequency magnetic fields deliberatively provided from the external system. Zaeimbashi et al. (2019) [79] introduced the NanoNeuroRFID system, based on FeGaB/AlN, designed for large-scale neural magnetic field transmission. Capable of supporting between 1000 and 10 000 devices, the system offers high spatial and temporal resolution while simultaneously harvesting energy to power-integrated circuits. To further allow safety, Choi et al. (2021) [69] developed a fully bioresorbable, battery-free, leadless cardiac pacemaker that operates via inductive charging. At the receiver coil’s resonance frequency (~13.5 MHz) with a transmitting voltage of 7 V, the device achieves a maximum output voltage of 13.2 V, demonstrating its potential for safe and efficient wireless cardiac pacing (Figure 6B). Gutruf et al. (2019) [70] developed a battery-free implantable device for multisite optical and electrical stimulation, eliminating the need for recharging or bulky battery packs. This design supports continuous operation while minimizing interference with natural animal behaviors (Figure 6C).

The giant magnetoelastic effect has recently emerged as a powerful mechanism for both energy harvesting and sensing, utilizing magnetoelastic materials coupled with conductive layers. This effect enables the conversion of mechanical deformation into electrical signals through magnetic induction, opening new possibilities for wearable and implantable self-powered systems. For instance, Zhao et al. (2021) [82] developed a textile-based magnetoelastic generator using NdFeB nanomagnets that combines magnetoelastic and magnetic induction effects. The device achieved a short-circuit current density of 0.63 mA cm^−2^ and an internal impedance of 180 Ω. Building on the same approach, Wang et al. (2022) [76] further demonstrated a magnetoelectric textile generator using NdFeB-based magnetic yarns, achieving a high power density of 3197 mW m^−2^ with a load resistance of 750 Ω. To improve the energy harvesting efficiency, Zhou et al. (2021) [71] introduced a soft magnetoelastic generator integrated into an implantable device for ultrasound excitation beneath porcine tissue, demonstrating its feasibility in biomedical applications (Figure 6D). Lastly, with combining remote powering and sensing together, Zhao et al. (2022) [81] applied the giant magnetoelastic effect in a soft, stretchable system to develop a self-powered implantable sensor for cardiac monitoring in a porcine heart model. The device exhibited stable electrical performance over extended ex vivo testing, highlighting its potential for long-term implantable use.

Another method of energy harvesting in implantable devices involves utilizing the motion of permanent magnets to induce current in conductive circuits. For example, Maharjan et al. (2019) [78] proposed a high-performance electromagnetic energy harvester featuring a rolling spherical magnet inspired by the cycloid curve. This design enables the magnet to follow the shortest-time trajectory along a curved path, maximizing the rate of magnetic flux change and, consequently, energy generation. Pancharoen et al. (2014) [84] developed a kinetic energy harvester intended for integration into a hip implant. A prototype was fabricated and tested, achieving a power output of 0.96 μW. More recently, Zhou et al. (2023) [77] introduced a flexible omnidirectional rotating magnetic array (FORMA), comprising miniaturized NdFeB magnetic balls embedded in PDMS. The FORMA system, combined with a flexible energy harvester, can be integrated into various implantable platforms to provide a compact power source. In addition to pure magnetic-based systems, alternative strategies such as photovoltaic harvesting have also been explored together with magnetic materials. Song et al. (2016) [72] demonstrated a flexible and implantable ultrathin photovoltaic energy harvester based on GaInP/GaAs. This system enables higher levels of in vivo energy harvesting and can be used to power medical implants or extend battery life by supplying supplemental energy whenever light is available, offering broad potential for biomedical applications (Figure 6E).

Magnetic field and materials have enabled a wide range of promising energy harvesting strategies toward wirelessly powered implantable biomedical devices and systems, such as inductive charging, magnetoelectric effect, and giant magnetoelastic effect. These strategies enable battery-free operation, device minimization, and extension in device longevity, thus supporting sustainable power delivery, wireless communication, and compatibility with deep-tissue and minimally invasive applications in complex *in vivo* environments.

## 6 | Discussion and Outlook

**FIGURE 7 |:**
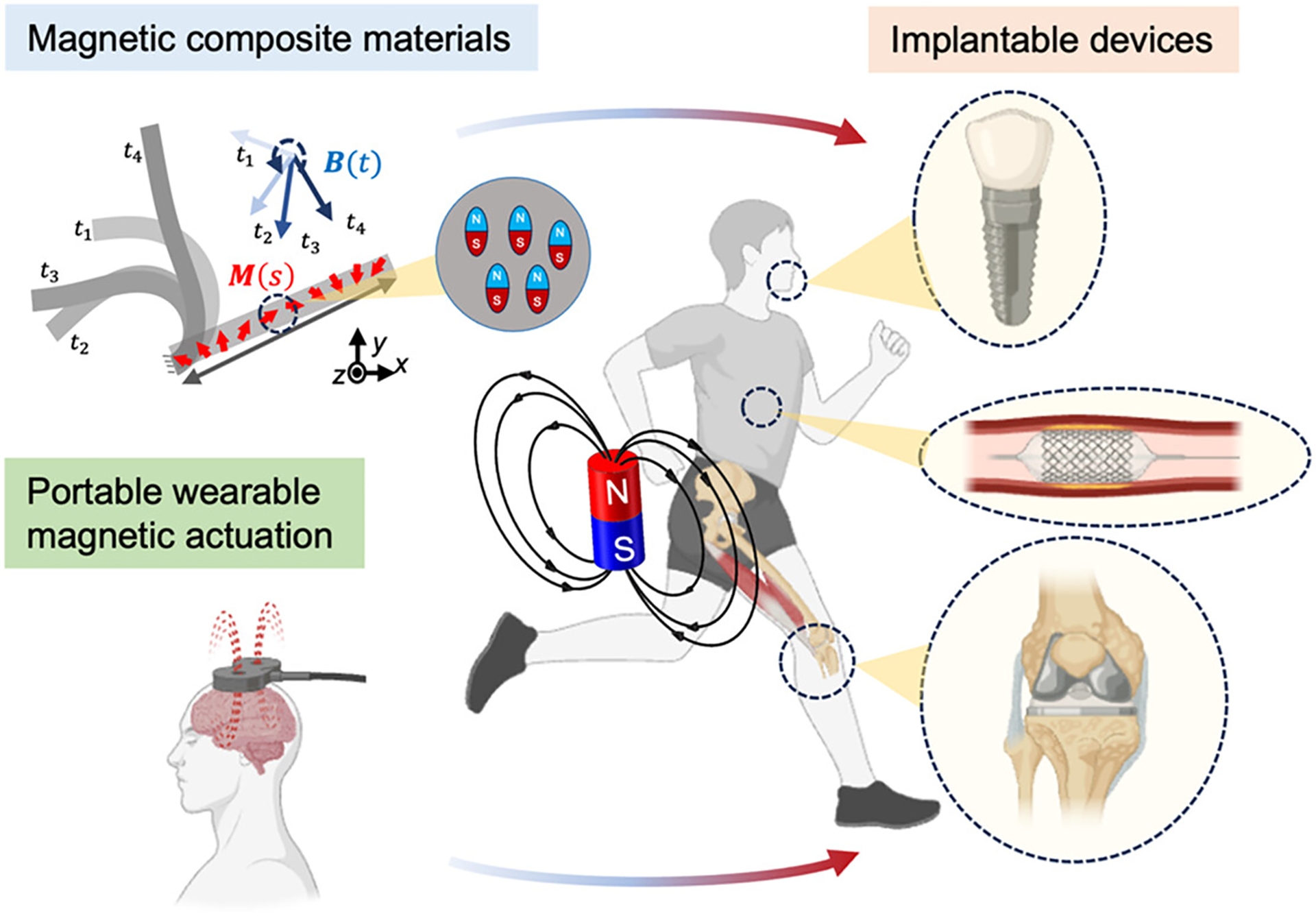
Outlook of magnetic materials-integrated implantable devices and their magnetic actuation system.

Magnetic fields and magnetic materials hold great promise for enabling miniaturized, fully wireless implantable devices that integrate sensing and actuation for robotic biomedical applications. Several key directions, as outlined in Figure 7, may be pursued to advance these technologies toward clinical translation.

### 6.1 | Biocompatibility

One of the primary concerns is ensuring that the magnetic materials are biocompatible, meaning they must not induce adverse immune responses, inflammation, or toxicity in the body. Some magnetic materials, such as certain metal oxides or alloys, may not be inherently biocompatible, releasing potentially harmful byproducts [85, 86]. Coating magnetic materials with biocompatible substances such as parylene-C or ceramics [87] and developing novel biodegradable magnetic materials are ongoing areas of research. Specific magnetic materials, particularly when used in nanoparticle form, can pose toxicity risks due to their ability to cross biological barriers or accumulate in certain tissues. This poses challenges for ensuring long-term safety, especially for devices intended to remain in the body for extended periods. Researchers are actively investigating biocompatible and biodegradable magnetic nanoparticles and coatings to mitigate these toxicity risks.

### 6.2 | Miniaturization

Many medical applications require extremely small devices, such as for minimally invasive surgeries or implanting sensors in narrow blood vessels [60] or other hard-to-reach areas. Miniaturizing magnetic components while maintaining functionality such as adequate magnetic field strength or sensor sensitivity is technically challenging. Advancements in nanomaterials and advanced manufacture such as combing 3D printing and micromolding [88] and other programing methods [89, 90], are helping to address miniaturization challenges by providing functional magnetic properties in smaller volumes.

### 6.3 | Magnetic Interference and Shielding

Magnetic materials can be influenced by external magnetic fields, including those from medical imaging devices like magnetic resonance imaging (MRI) machines, leading to potential interference or malfunction of implantable devices. This can pose a safety risk or compromise the device’s therapeutic functions. Designing devices with built-in shielding to block or reduce the effects of external magnetic interference is critical. Additionally, developing materials and implantable devices that are MRI-compatible [63] is an ongoing area of research.

### 6.4 | Controlled Actuation and Selectivity

Precisely controlling the actuation of magnetic materials inside the body is difficult, particularly for tasks like drug delivery, tissue regeneration, or remote control of implants. The selective and precise manipulation of magnetic fields without affecting surrounding tissues or inducing unwanted side effects remains a significant obstacle. More sophisticated magnetic field control systems are being explored, such as using gradient magnetic fields, advanced external control systems, or materials that respond to specific magnetic field strengths [91], frequencies [92], and heterogeneous magnetic field distribution [93].

### 6.5 | Degradation and Biodegradability

The degradation of magnetic materials within the body can lead to the release of toxic byproducts or trigger inflammatory responses. However, in certain applications such as temporary drug delivery systems or biodegradable sensors [7], controlled degradation is desirable once the device has fulfilled its function. Current research focuses on engineering magnetic materials that either degrade into biocompatible, nontoxic byproducts or remain stable and inert throughout their intended lifespan. Long-term implantable devices, such as pacemakers, neural stimulators, or orthopedic implants, must maintain functionality for several years. Over time, magnetic materials may experience corrosion, mechanical wear, or other forms of degradation that compromise device performance or lead to failure. To address these challenges, researchers are developing corrosion-resistant magnetic materials, durable coatings, and robust encapsulation strategies to enhance the long-term stability and reliability of implantable systems.

### 6.6 | Wireless Communications

Radio frequency identification (RFID) technology utilizes electromagnetic fields to automatically identify and track tags attached to objects. In implantable medical devices, RFID tags can be employed to sense and wirelessly transmit physiological data such as temperature, pressure, or biochemical markers. These tags can be either passive (powered by an external reader) or active (battery-powered). Applications include monitoring intraocular pressure, glucose levels, and tracking the position or status of implants. A notable subset of RFID is near-field communication (NFC), which enables short-range data exchange between devices. NFC-powered implants, typically passive, can transmit sensor data when in proximity to an external reader or smartphone. Common applications include glucose monitoring, controlled drug delivery, and patient identification. However, the sensing distance needs to be significantly increased for deep tissue implantable devices. Alternative strategies include distribute the communication module and the sensor module at separate locations [94] to allow the communication module close to the surface of the skin.

### 6.7 | Integration with Other Medical Technologies

Many implantable devices must interface seamlessly with existing medical technologies, including imaging systems such as MRI, requiring MRI-compatible designs that ensure safety within high-field environments [95, 96]. Equally important is the minimally invasive delivery of these devices using standard clinical tools such as endoscopes and bronchoscopes. Achieving safe and effective integration of magnetic materials within these procedures presents notable challenges. However, recent advances in flexible electronics, microfluidics, and multimaterial fabrication techniques offer promising strategies to enhance compatibility, functionality, and overall device performance. Importantly, the design of implantable systems should simultaneously consider both their intended clinical function and their compatibility with established medical workflows.

### 6.8 | Clinical Potential

Magnetic material-enabled implantable robotic devices hold significant clinical potential by enabling wireless, minimally invasive therapies that integrate sensing, actuation, and energy transfer. These devices can deliver drugs on-demand, monitor physiological parameters in real time, and be remotely actuated or navigated through complex anatomical regions without tethered components. Applications range from targeted drug delivery [23, 38] and responsive orthopedic implants to smart stents [34, 57] and magnetic microsurgical tools. Their programmability and soft, biocompatible designs make them especially suited for dynamic biological environments. Continued advances in miniaturization, biocompatibility, and magnetic field control are key to translating these technologies into clinical use.

## Figures and Tables

**TABLE 1 |:** Representative magnetic materials used in implantable devices.

Type of materials	Example	Unique property	Application in implantable devices	References
Ferromagnetic	Neodymium–Iron–Boron alloy	Large remanence	Magnet-based tracking, actuation	[9, 23]
Superparamagnetic	FePt alloy	Biocompatible, large magnetization	Hyperthermia, drug carrier	[24, 25]
	Iron oxide nanoparticles	Biocompatible	Drug carrier	[26–28]
	Ferrofluid	Liquid	Sensing soft tissue	[29, 30]
Magnetoelectric	Magnetic-PZT composite	Electrical effect	Stimulation, energy harvesting	[18]
Magnetoelastic	Magnetic-elastomer composite	Shape-morphing	Pump, valve, sensing	[12, 13, 31]

**TABLE 2 |:** Magnetic material for actuation in implants.

Function	Device form	Implanted location/applications	Material	Actuation field	In vivo test	References
Pump	Stent	Stent, esophagus/dysmotilityof the esophagus	NdFeB-polymer composite	40–60 mT	No	Sharma et al. 2024 [33]
Pump	Stent	Stent, trachea, airway mucus clogging	NdFeB-polymer composite	~40 mT	No	Wang et al. 2023 [34]
Pump	Insulin pump	Skin, diabetes	Magnets	~100 mT	Yes	Lee et al., 2017 [35]
Pump	Surface actuator	Bladder/underactive bladder	NdFeB-polymer composite	~300 mT	Yes	Yang et al. 2022 [36]
Pump	Catheter	Brain hemorrhage	Nickel	15 mT	Yes	Yang et al., 2022 [37]
Valve	Dental implant	Tooth/drug administration	NdFeB-polymer composite	<65 mT	No	Xu et al., 2024 [23]
Valve	Intravitreal drug delivery system	Eye/Retinal neovascularization	Iron oxide-polymer composite	~200 mT	Yes	Wang et al., 2018 [38]
Valve	Glaucoma drainage devices	Eye/Glaucoma	SIBS-carbonyl iron composite	270 mT	No	Pereira et al. 2023 [39]
Valve	Soft valve	Esophagus, stomach/gastroesophagealreflux disease	NdFeB magnets	0 mT	Yes	Ganz et al., 2008 [40],
Liquid biopsy	Soft capsule	GI tract	NdFeB-polymer composite	50–70 mT	No	Dong et al., 2024 [14]
Drug delivery	Triboelectric nanogenerator	Cancer	Magnets	Not available	Yes	Zhao et al., 2019 [41]
Cell delivery	Particlesmicrocarrier system	Tissue regeneration	Iron oxide-polymer composite	50 mT	No	Go et al., 2021 [42]
Drug delivery	Tubular microrobots	Eye	CoNi	40 mT	Yes	Chatzipirpiridis et al., 2015 [43]
Drug delivery	Capsule	GI tract	Magnets	60–140 mT	Yes	Zheng et al., 2023 [44]
Hyperthermia	Implantintegrated trapped device	Blood vessel/tumor cell removal	Coil	22.5 kAm^−1^	Yes	Yin et al., 2022 [45]
Hyperthermia	Nanofiber	Tumor	Iron oxide-polymer composite	12.57 kAm^−1^	No	Sasikala et al., 2015 [46]
Drug delivery	Liquid metal (EGaln)	Cancer therapy	Liquid metal (EGaln)	9.6 kAm^−1^	Yes	Wang et al., 2019 [47]
Stimulation	Microcoil	Cortex	Copper coil	49 kVm^−2^	Yes	Lee et al., 2016 [48]
Stimulation	Microcoil	Cortex	Copper coil	156–528 mT	Yes	Lee et al., 2024 [49]

**TABLE 3 |:** Magnetic material for sensing in implantable devices.

Sensing property	Implanted location	Materials	Sensing mechanism	Sensitivity	Sensing range	In vivo	References
Motion	Spine and joint	Fe_3_O_4_-polymer composite	Superparamagneticity	2.2 μV/degree	0–150 degrees	Yes	Shi et al., 2024 [54]
Motion	Knee	Magnets	Magnetic sensor	N/A	1.6 –73.61 degrees	No	Arami et al., 2013 [55]
Tissue viscoelasticity	GI tract	NdFeB-polymer composite	Dynamic robot-tissue interaction	0.0006 kPa^−1^	1.9–51.2 kPa	No	Wang et al., 2023 [15]
Biofluid viscosity	Brain	Maget	Vibration	24.79 cP^−1^	3.5–4.5 cP	Yes	Wan et al., 2024 [56]
Biofluid viscosity	GI tract	NdFeB-polymer composite	Magnetic actuation	1.3 mT/(Pas)	2.7–17.9 Pas	No	Xiao et al., 2023 [16]
Biofluid viscosity	Trachea	NdFeB-graphene-polymer composite	Fluid-structure interaction	0.05 Pa^−1^s^−1^	0–10 Pas	No	Wang et al., 2024 [57]
Glucose	Brain	Maget	Vibration	0.1 Hz/mM^−1^	2–30 mM	Yes	Wan et al., 2024 [56]
Pressure	Brain	Maget	Vibration	4.44 Hz kPa^−1^	3–5 kPa	Yes	Wan et al., 2024 [56]
Pressure	Tissue	Inductive coupling	Strain gauge	0.0095 MHz/mmHg	0–40 mmHg	Yes	Lin et al., 2024 [58]
Pressure	Artery	Inductive coupling	Strain gauge	0.005/kPa	20–450 kPa	Yes	Boutry et al., 2019 [59]
Pressure	Artery	Inductive coupling	Strain gauge	0.013 kPa - 1	0–110 mmHg	Yes	Herbert et al., 2022 [60]
pH	GI tract	NdFeB-polymer composite	Adhesion-based	1 pH	2–9 pH	No	Wang et al., 2023 [15]
Pulse rate	Artery	Inductive coupling	Strain gauge	N/A	N/A	Yes	Herbert et al., 2022 [60]
Flow	Artery	Inductive coupling	Strain gauge	0.04 Hz.min ml^−1^	0–1000 ml min^−1^	Yes	Herbert et al., 2022 [60]
Biofluid volume	Trachea	NdFeB-polymer composite	Capacitive measurement	40 pF mm^−1^	0–5 mm	No	Wang et al., 2024 [57]
Magnetic resonance relaxometry	Heart	Iron oxide nanoparticle	Magnetic resonance relaxometry	10–100 ng ml^−1^	100 ng ml ^−1^–1 μg ml^−1^	Yes	Ling et al., 2011 [61]

**TABLE 4 |:** Magnetic material for energy harvesting.

Type	Materials	Range	Size	Power	References
Magnetoelectric	PZT/Metglas	Within 3 cm	1.75 mm × 5 mm Thickness: 0.3 mm	Peak power: 1.17 mW	Chen et al., 2022 [68]
Magnetoelectric	Nickel-coated lead zirconate titanate (PZT)/Metglas	Within 30 mm	Total volume: 8.3 mm^6^	Maximum output: 23.7 μW	Yu et al., 2020 [52]
Magnetoelectric	PZT/Metglas	Within 40 mm	6.2 mm^3^	3.5 V	Yu et al., 2022 [73]
Magnetoelectric	NdFeB	N/A (Self-powered)	1.70 in^3^	0.4 mW	Nasiri et al., 2011 [74]
Magnetoelectric	NdFeB	Within 20 mm	250 mm^3^	0.96 μW	Pancharoen, et al., 2014 [75]
Electromagnetic	Magnetic yarns with NdFeB particles	Within 30 mm	5 × 5 cm^2^ (Magnetic fabric), 500 turns	Power density: 3197 mW m^−2^	Wang et al., 2022 [76]
Electromagnetic	Rotating NdFeB magnetic array	Within 12 mm	16 mm by 16 mm by 4 mm	Maximum current density of 2.27 × 10^6^ A/m^2^	Zhou et al., 2023 [77]
Electromagnetic	Spherical NdFeB magnet	Within 6 mm	10 mm in diameter	Peak power of 74 mW (5 Hz, 104.7 Ω)	Maharjan et al., 2019 [78]
Photovoltaic	GaInP/GaAs,	Within 750 μm	Area: 6.8 mm^2^	Maximum power density: 22 mW cm^−2^	Song et al., 2016 [72]
Inductive	Rx coil: FeGaB/AlN	Within 2 mm	0.006 mm^−2^, thickness: 500 nm	Not available, Voltage: 1.2 V	Zaeimbashi et al., 2019 [79]
Inductive	Tx coil: Tungsten-coated magnesiumRx coil: Copper	Within 17 cm	Tx coil diameter: 64 mm, Rx coil diameter: 25 mm	Not available, Voltage: 13.2 V	Choi et al., 2021 [69]
Inductive	Rx coil: Ti/Pt	Within 5 cm	Length: 3 cmPt layer thickness: 200 nm	10 mW mm^−2^	Gutruf et al., 2019 [70]
Inductive	Rx coil: Cooper	Within 4 cm	13 mm × 8 mm × 3 mm	Maximum output: 24 mW	Sivaji et al., 2019 [53]
Inductive	Rx coil: PI/Au	Within 5.5 cm	Radius: 5mm, Thickness: 0.3 mm	Not available	Herbert et al., 2022 [60]
Inductive	Rx coil: Liquid metal (LM) [eutectic gallium-indium (EGaIn)]	Within 20 cm	Diameter: 2cm, Length: 12 cm	111.51 mW	Zhang et al., 2023 [80]
Giant magnetoelastic	Micromagnets (MQFP/NdFeB) and a porous silicone rubber/Copper	Within 5 mm	40 × 40 mm^2^	Peak power: 30.69 μW /0.94 mA	Zhou et al., 2021 [71]
Giant magnetoelastic	NdFeB and Liquid metal (Ga/In)	N/A (Self-powered)	5 mm × 5 mm	Not available, Current: 10 μA	Zhao et al., 2022 [81]
Giant magnetoelastic	Fiber with NdFeB nanomagnets	N/A (Self-powered)	4 by 6 cm, using fibers that have a cross-section area of 12 mm^2^	Peak power: 16 mW	Zhao et al., 2021 [82]
Giant magnetoelastic	Metglas	Within 30 mm	Length: 30 mm Thick: 30 μm	Not available, Voltage: 2 V	Mouzakis et al., 2008 [83]
